# Distribution and Structure of Synapses on Medial Vestibular Nuclear Neurons Targeted by Cerebellar Flocculus Purkinje Cells and Vestibular Nerve in Mice: Light and Electron Microscopy Studies

**DOI:** 10.1371/journal.pone.0164037

**Published:** 2016-10-06

**Authors:** Hitomi Matsuno, Moeko Kudoh, Akiya Watakabe, Tetsuo Yamamori, Ryuichi Shigemoto, Soichi Nagao

**Affiliations:** 1 Laboratory for Motor Learning Control, Riken Brain Science Institute, Hirosawa 2-1, Wako, Saitama, 351-0198, Japan; 2 Laboratory for Molecular Analysis of Higher Brain Function, RIKEN Brain Science Institute, Hirosawa 2-1, Wako, Saitama, 351-0198, Japan; 3 Division of Cerebral Structure, National Institute for Physiological Sciences, 5-1 Higashiyama, Myodaiji, Okazaki, Aichi, 444-8787, Japan; Universidad de Salamanca, SPAIN

## Abstract

Adaptations of vestibulo-ocular and optokinetic response eye movements have been studied as an experimental model of cerebellum-dependent motor learning. Several previous physiological and pharmacological studies have consistently suggested that the cerebellar flocculus (FL) Purkinje cells (P-cells) and the medial vestibular nucleus (MVN) neurons targeted by FL (FL-targeted MVN neurons) may respectively maintain the memory traces of short- and long-term adaptation. To study the basic structures of the FL-MVN synapses by light microscopy (LM) and electron microscopy (EM), we injected green florescence protein (GFP)-expressing lentivirus into FL to anterogradely label the FL P-cell axons in C57BL/6J mice. The FL P-cell axonal boutons were distributed in the magnocellular MVN and in the border region of parvocellular MVN and prepositus hypoglossi (PrH). In the magnocellular MVN, the FL-P cell axons mainly terminated on somata and proximal dendrites. On the other hand, in the parvocellular MVN/PrH, the FL P-cell axonal synaptic boutons mainly terminated on the relatively small-diameter (< 1 μm) distal dendrites of MVN neurons, forming symmetrical synapses. The majority of such parvocellular MVN/PrH neurons were determined to be glutamatergic by immunocytochemistry and in-situ hybridization of GFP expressing transgenic mice. To further examine the spatial relationship between the synapses of FL P-cells and those of vestibular nerve on the neurons of the parvocellular MVN/PrH, we added injections of biotinylated dextran amine into the semicircular canal and anterogradely labeled vestibular nerve axons in some mice. The MVN dendrites receiving the FL P-cell axonal synaptic boutons often closely apposed vestibular nerve synaptic boutons in both LM and EM studies. Such a partial overlap of synaptic boutons of FL P-cell axons with those of vestibular nerve axons in the distal dendrites of MVN neurons suggests that inhibitory synapses of FL P-cells may influence the function of neighboring excitatory synapses of vestibular nerve in the parvocellular MVN/PrH neurons.

## Introduction

The horizontal vestibulo-ocular reflex (HVOR) and optokinetic response (HOKR) are respectively compensatory eye movements for the movement of the head and external visual surrounding on the horizontal plane. These two ocular reflexes have been studied as an experimental model of motor learning, because their dynamic characteristics are modifiable by training that induces motor error. For example, when animals are trained to watch the motion of a stripe- or dot-patterned screen, with or without the motion of animals, the mismatch between the screen and eye motion is sensed as retinal slips, which induce adaptation in the magnitude of eye movements evoked by HVOR or HOKR (e.g., [1]). In the neural circuitry of HVOR and HOKR, both the cerebellar flocculus (FL) and the medial vestibular nucleus (MVN) targeted by FL (FL-targeted MVN) are the major players (e.g., [2]), but their roles in adaptation have been an issue of debate for more than two decades [3–7]. Note that FL Purkinje cells (P-cells) directly inhibit the MVN neurons relaying HVOR and HOKR, not via the cerebellar nuclear neurons. Now, increasing lines of evidence have been accumulated from experiments of mice [8,9], cats [10], and monkeys [11], suggesting that the memory trace of short-term adaptation that decays within 24 h is maintained in FL, whereas the memory trace of long-term adaptation that remains for more than 24 h is maintained in FL-targeted MVN.

The location and responsiveness of FL Purkinje cells (P-cells) involved in HVOR and HOKR adaptations have been examined in mice [12], rabbits [13–16], and monkeys [17, 18]. Moreover, two recent electron microscopy (EM) studies of mice [19,20] have demonstrated quantitatively that the density of α-amino-3-hydroxy-5-methyl-isoxazalone-propionic acid (AMPA)-type glutamate receptors at FL parallel fiber-P-cell synapses decreases in parallel with the short-term HOKR adaptation without affecting any other synaptic structures, suggesting that the long-term depression of FL P-cell synapses underlies adaptation [2,6,7]. In contrast, we have a relatively limited knowledge on the role of FL-targeted MVN neurons. A single-unit recording study of rabbits [21] revealed that FL-targeted MVN neurons located in the magnocellular and parvocellular MVNs receive both vestibular and visual inputs and project to extraocular muscle motor neurons. However, such FL-targeted MVN neurons were only a small fraction of MVN neurons in the studies of cats [22] and mice [23], and their synaptic structures have not been compared sufficiently between the magnocellular and parvocellular MVNs.

In this study, we injected the anterograde tracer, green fluorescent protein (GFP)-expressing lentivirus vector, into FL and the anterograde tracer, biotinylated dextran amine (BDA), into the horizontal semicircular canal in mice. We then examined the spatial distribution of synapses of FL P-cells and those of vestibular nerve in MVN neurons by light microscopy (LM) and EM. The results suggest that the inhibitory synapses of FL P-cell axons and the excitatory synapses of vestibular nerve axons are often located close to each other on the distal dendrites of FL-targeted MVN neurons, specifically in the parvocellular MVN/ prepositus hypoglossi (PrH) border region.

## Materials and Methods

### Ethics statement

The experimental protocols followed the principles of laboratory animal care (NIH publication No. 86–23, revised in 1996) and were approved by the RIKEN Wako Animal Experiments Committee and RIKEN Wako Genetic Recombinant Experiment Safety Committee (approval numbers: H18-2B015, H20-2-221, H22-2-225, H24-2-224, H28-2-222, 2005–071, 2008–047, 2011–037, and 2014-062(11)).

### Animals

C57BL/6J male mice (8–11 weeks old) were obtained from Japan SLC (Shizuoka, Japan). Thy1-GFP M-line transgenic mice were kindly provided by Dr. Joshua Sanes (Harvard University). We used male conventional (non-transgenic) C57BL/6J mice except for LM experiments showing the distribution of P-cell axonal boutons on the MVN dendrites. All efforts were made to minimize the number of mice used and their suffering throughout the course of experiments.

### Lentivirus preparation

Vesicular stomatitis virus-G protein (VSVG) pseudotyped lentivirus were produced as described in a previous report [24]. Plasmid vectors were provided by St. Jude Children’s Research Hospital [25]. The expression plasmid (pCL20c MSCV-GFP) was cotransfected into HEK293T cells [26] with two packaging plasmids (pCAGkGP1.1R and pCAG4-RTR2) and VSVG-expressing plasmid (pCAGGS-VSV-G) by the calcium phosphate precipitation method. The viral vector was harvested 40 h after transfection, and concentrated by ultracentrifugation.

### Tracer injection

Under isoflurane (Escain, Mylan-Japan, Tokyo, Japan) anesthesia and aseptic conditions, the mice were fixed to a standard mouse stereotaxic apparatus. Tiny holes were opened on the temporal bones over the cerebellar paraflocculus using a surgical drill under a surgical microscope. A glass micropipette (tip diameter, ~20 μm) connected to a microsyringe (Hamilton, 7012) mounted on a standard micromanipulator was inserted into FL via the paraflocculus. The lentivirus vector (0.1–0.25 μl) was slowly injected manually into bilateral FLs, taking at least 30 min for each. To further trace the vestibular nerve axons, the three mice infected with the GFP-expressing lentivirus vector received subsequent BDA injections in the vestibular organ under isoflurane anesthesia. After opening the tympanic membrane and removing the auditory ossicles, the superior part of the exit point of the facial nerve was drilled to open a small hole on the ampulla of the horizontal semicircular canal. Several pieces of gelform sponge (Astellas, Tokyo, Japan) soaked with 10% BDA (Sigma Aldrich, St Louis, MO, USA) dissolved in saline were inserted into the hole made on the horizontal canal after suctioning the lymphatic fluid. Three to four days after BDA labeling, the mice were sacrificed by perfusion fixation.

### Histology and image analysis

Under pentobarbital anesthesia (i.p., 60 mg/kg body weight, Nembutal, Dainippon- Sumitomo Pharma, Osaka, Japan), mice were perfused transcardially with 50 ml of 0.1 M phosphate buffered saline (PBS), followed by 50 ml of 4% paraformaldehyde (Merck Japan, Tokyo, Japan) in 0.1 M phosphate buffer. After perfusion, the brain of each mouse was extracted from the skull, postfixed in the same fixative overnight at 4°C, and then cryoprotected in 30% sucrose overnight at 4°C. Serial coronal sections (thickness, 40 μm) were prepared using a freezing microtome, then blocked in 5% goat serum in PBS containing 0.25% TritonX-100 (Nacalai Tesque, Kyoto, Japan), and incubated with the primary antibodies overnight at 4°C as described below.

To trace the axons of FL P-cells in MVN, the sections were reacted with rat anti-GFP antibody (1:1000, GF090R, Nacalai Tesque) and mouse anti-NeuN antibody [1:200, MAB377, Chemicon (Merck Japan, Tokyo, Japan)], washed with PBS, and then incubated with secondary antibodies Alexa 488 goat anti-rat IgG and Alexa 555 goat anti-mouse IgG [1:300, Molecular Probes (Thermo Fisher Scientific), Waltham, MA, USA] for 1 h. To investigate the spatial relationship between FL P-cell axonal boutons and vestibular nerve axonal boutons on MVN dendrites, sections of four Thy1-GFP M-line transgenic mice [27], which were injected with BDA into the horizontal semicircular canal 4–8 days previously, were incubated with rat anti-GFP antibody (1: 1000, GF090R, Nacalai Tesque) and rabbit anti-protein kinase Cγ-subunit (PKCγ) antibody (1:50, P-3328, Sigma Aldrich). The reaction was then visualized using Alexa 488 goat anti-rat IgG, Alexa 555 goat anti-rabbit IgG, and Alexa 647-conjugated streptavidine (1:300, Molecular Probes). For labeling the synapses between P-cells and MVN neurons, we performed triple immunostaining of some sections from two thy1-GFP mice with rat anti-GFP antibody, rabbit anti-PKCγ antibody, and mouse anti-synaptophysin antibody (1:100, SY38, Chemicon), and the signals were visualized with Alexa fluor dye-conjugated secondary antibodies.

To identify the type of neurotransmitters of MVN neurons that receive FL axons on the dendrites, we fixed one Thy1-GFP M-line transgenic mouse with mixture of 0.5% paraformaldehyde and 2.5% glutaraldehyde (Nacalai Tesque) in 0.1 M phosphate buffer, postfixed on ice for 15 min, and performed double staining using anti-GFP antibody and either one of three antibodies against neurotransmitters [anti-glutamate (anti-Glu, 1:1000, G-6642, Sigma Aldrich), anti-glycine (anti-Gly, 1:500, AB139, Chemicon), and anti-γ-amino-butyric acid (anti-GABA, 1:1000, A-2052, Sigma Aldrich)].

Fluorescence images of these sections were obtained using a Fluoview FV1000 confocal microscope (Olympus, Tokyo, Japan) or a digital fluorescence BZ-9000 microscope (KEYENCE, Tokyo, Japan). To quantify the distribution of P-cell axonal projections, we obtained z-stacked high-magnification images of three coronal sections with 160 μm interslice distance, using a 40x oil immersion objective lens to detect smaller axonal varicosities. GFP-labeled axonal terminals or varicosities were identified as synaptic boutons and their number was counted manually per 20 μm x 20 μm grid on the z-projection images. The fluorescent images obtained from 4 injection cases at the same coordinate in the brain atlas [28] were overlapped to calculate the averaged axonal density per square grid, which was represented by heat maps of MVN. For colocalization experiments, we used sections of one thy1-GFP M-line mouse, and randomly obtained the confocal images of GFP- labeled neurons that extend their dendrites to the parvocellular MVN/PrH. We collected at least more than 20 GFP- labeled cells from coronal sections for the staining of each antibody. We then counted the numbers of neurons showing double-positive staining for GFP and for one of the three types of neurotransmitter. Golgi staining was performed using three C57/BL6J mice in accordance with the manufacturer’s protocol (Rapid GolgiStain Kit, FD NeuroTechnologies, Columbia, MD, USA).

### Immunoelectron microscopy

Post-perfusion fixed brains obtained from one C57/BL6J mouse, which received FL lentivirus injection, and two C57/BL6J mice, all received FL lentivirus and BDA vestibular organ double injections, were postfixed with 0.05% glutaraldehyde and 4% paraformaldehyde overnight at 4°C, and 50-μm-thick coronal sections were prepared using a vibratome (Leica Microsystems, Wetzlar, Germany). The sections were initially incubated with the anti-GFP mouse antibody (1:3000, mFX73, WAKO, Tokyo, Japan) for two days at 4°C and then incubated with biotinylated anti-mouse IgG (1:100; Vector Laboratories, Burlingame, CA, USA). Finally, they were visualized by avidin-biotin peroxidase complex (ABC)–diamonobenzidine (DAB) reaction. For pre-embedding double labeling, sections were incubated with rabbit anti-GFP (1:500) [29] for two days at 4°C, and then incubated with gold-particle-conjugated streptavidin (1:100, GAR-811211/1, Aurion, Wageninge, The Netherlands) for 3 h. The gold particles were enhanced with a silver enhancement kit (Nanoprobes, Yaphank, NY, USA). Sections were further incubated with the biotinylated anti-rabbit secondary antibody (1:100, Vector Laboratories) overnight at 4°C followed by ABC—DAB reaction. After osmification, the stained sections of the dorsal MVN area, which contains the border region between the parvocellular MVN and PrH (approximately 200 μm below the forth ventricle) were flat-embedded in epoxy resin. Ultrathin sections (thickness, 0.07 μm) were contrasted with uranyl acetate/lead citrate and analyzed by transmission EM (JEM 1010 and 1200ex, JEOL, Tokyo, Japan). We used μm scales to present the length of dendritic diameter throughout this article.

## Results

### Anterograde tracing of FL P-cell axons by lentiviral injections

**Fig 1:**
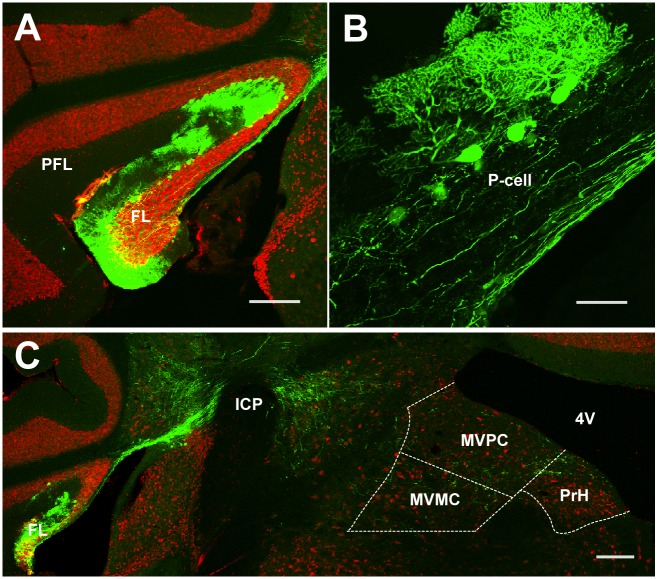
Anterogradely labeled FL P-cell axons in the medial vestibular nucleus (MVN) and prepositus hypoglossi (PrH) in case (#) 94R. **A**, GFP expression in FL at 3 weeks after lentiviral injection. Sections were stained with anti-GFP (green) and neuronal marker anti-NeuN (red) antibodies. **B**, GFP was preferentially expressed in FL P-cells. **C**, GFP-labeled axons of FL P-cells in the ventromedial MVN. FL, flocculus; icp, inferior cerebellar peduncle; MVMC, magnocellular MVN; MVPC, parvocellular MVN; P-cell, Purkinje cell; PFL, paraflocculus; PrH, prepositus hypoglossi nucleus; 4V, fourth ventricle. Scale bars, 200 μm (**A** and **C**) and 50 μm (**B**).

We injected GFP-expressing VSVG pseudotyped lentivirus into bilateral FLs in 11 C57/BL6J mice (13 injection cases, Table 1). The injection sites covered the middle zone of FL, where P-cells project axons to MVN in rabbits [13], cats [30], mice [12], and monkeys [17,31]. After the injections, the mice were reared in their home cage for 2–3 weeks without showing any sign of ataxia. In brain sections obtained from these mice, a strong GFP signal was detected in the P-cells of the middle zone of FL (Fig 1A, case (#) 94R). A weak GFP signal was also observed in the adjacent paraflocculus (PFL), which was presumably due to the diffusion of the virus from FL.

**Table 1:** Labeled Purkinje cell axonal boutons after FL lentivirus injections.

Case	Injection site	Boutons in MVN			Other vestibular nuclei
		MVMC	MVPC	PrH	
6R	whole FL, >10 neurons in PFL	+++	++++	++++	LV, SuV, y
12L	whole FL, a few neurons in PFL	+++	++++	+++	LV, SuV, y
14R	FL (zones 2 and 3), a few neurons in PFL	++	++	+	LV, SuV
14L	whole FL, a few neurons in PFL	+++	++++	+++	LV, SuV, LatPC
15R	whole FL	+++	+++	++	LV, SuV, LatPC
16L	FL (zones 1, 2, and 3)	+	+++	+++	LV, SuV, y
24R	whole FL, a few neurons in PFL	++	+++	++++	LV, SuV, y
71L	FL (zones 2, 3, and 4), a few neurons in PFL	++++	++	+++	LV, SuV
74R	whole FL	+++	++++	++++	LV, SuV, y
82R	whole FL, >10 neurons in PFL	++	+++	+++	LV, SuV
94R	whole FL, a few neurons in PFL	++++	++++	++++	LV, SuV
94L	whole FL, >10 neurons in ventral PFL	+++	++++	++++	LV, SuV
95R	FL (zones 2, 3, and 4), a few neurons in PFL	++++	+++	+	LV, SuV

Density of labeled boutons for each subregion were evaluated for two brain sections for each case (approximately 6.00 mm and -6.36 mm from bregma) as +, 0–50 boutons; ++, 50–100 boutons; +++, 100–200 boutons; ++++, >200 boutons. FL, flocculus; LatPC, parvocellular region of lateral cerebellar nucleus; LV, lateral vestibular nucleus; MVN, medial vestibular nucleus; MVMC, magnocellular MVN; MVPC, parvocellular MVN; PFL, paraflocculus; PrH, prepositus hypoglossi nucleus; SuV, superior vestibular nucleus; y, group y. Injection sites in FL are shown as zones 1~4, according to [12], in which FL was divided to four sagittal zones in the lateral-ventral to medial-dorsal direction. Labeled neurons in PFL were counted at the level of the injection site.

FL P-cells were labeled selectively with no retrogradely labeled neurons in MVN or FL, as reported in previous studies in which the VSVG pseudotyped lentiviral vector was used as the anterograde tracer in the cerebral cortex [32] and hippocampus [33]. Furthermore, somata, dendritic branches, and axons of P-cells were clearly labeled by GFP (Fig 1B and 1C). GFP was also expressed in some of Bergman’s glia cells (not shown in figures) in FL, but very rarely in granule cells and other interneurons that innervate FL P-cells. In the analysis of the projection pattern of FL P-cells, we excluded the cases in which the GFP expression level in PFL was higher than that in FL from the analysis.

The anterogradely labeled FL P-cell axons projected initially dorsally, then turned ventromedially at the dorsal part of the inferior cerebellar peduncle (icp), and entered into the ipsilateral MVN (Fig 1C). In all the injection cases, the labeled axons were distributed both in the magnocellular MVN and parvocellular MVN regions (Fig 1C). Labeled FL P-cell axons or axonal boutons were frequently observed in PrH and other vestibular nuclei (Table 1).

### Distribution of FL P-cell axonal boutons in MVN

**Fig 2:**
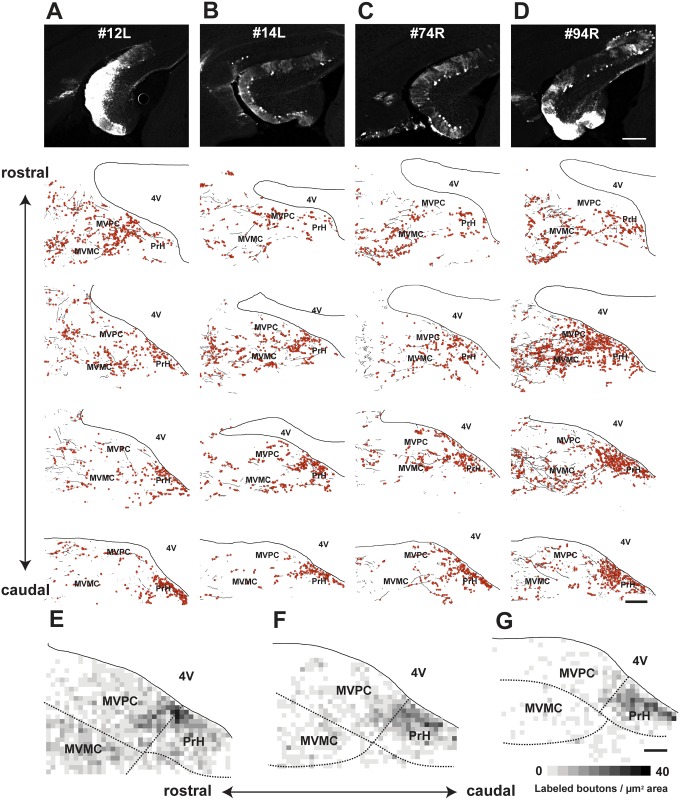
Distribution of FL P-cell axons and their boutons in MVN/PrH after FL lentivirus injections. **A**–**D**, Tracing of anterogradely-labeled FL axons in four mice. Despite the difference of density of GFP expression among four cases shown in the photographs in the top row, labeled FL P-cell axons were distributed throughout MVN and PrH in all of the four cases. Distributions of the labeled axons in MVN and PrH for each injection are summarized in 4 images that are shown from the rostral to caudal MVN/PrH. **A**, case (#) 12L; **B**, #14L; **C**, #74R; **D**, #94R. Labeled FL P-cell axons and their boutons are respectively shown by black lines and red circles. **E**–**G**, Summary of distribution of FL P-cell axonal boutons at rostral (**E)**, middle (**F)**, and caudal (**G**) MVN. The averaged density of labeled axonal boutons was calculated from cases 12L, 14L, 74R, and 94R. Although the FL P-cell axonal boutons were observed throughout MVN, they were densely present in the medial part of parvocellular MVN/PrH. MVMC, magnocellular MVN; MVPC, parvocellular MVN; PrH, prepositus hypoglossi nucleus; 4V, fourth ventricle. Scale bars, 200 μm (**A**–**D**) and 100 μm (**E**–**G**).

The GFP-labeled FL P-cell axonal boutons were widely distributed in the magnocellular MVN, parvocellular MVN, and PrH along the rostrocaudal axis (Fig 2A–2D) in coronal sections at approximately –6.0 to –6.5 mm from the bregma [28]. To quantitatively map the distribution of FL P-cell axonal boutons in MVN, we obtained the average density of labeled axonal boutons per unit area (Fig 2E–2G, cases 12L, 14L, 74R, and 94R) on three sequential sections for each case.

The labeled FL P-cell axonal boutons were distributed in both the magnocellular MVN and the border region between the parvocellular MVN and PrH, with a slight predominance in the parvocellular MVN/PrH (Fig 2E–2G and Table 1). The distribution of the axonal boutons of FL P-cells in the magnocellular MVN was similar to that found in a previous study using the transgenic mice that specifically expressed tau-GFP in P-cells [23].

**Fig 3:**
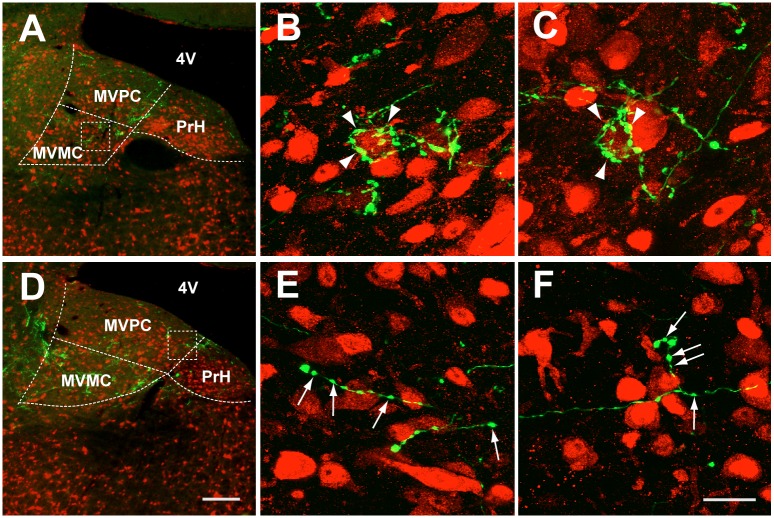
Two distinct types of FL P-cell axonal boutons observed in MVN/PrH (case (#) 94R). **A**–**C**, Merged images of GFP-fluorescent FL P-cell axonal boutons (green) and anti-NeuN-labelled somata (red) of MVN neurons. In **A**, anterogradely labeled FL P-cell axonal boutons were observed in the magnocellular MVN, in which the somata and proximal dendrites of MVN neurons were surrounded by a small group of axons, showing basket like structures (arrowheads in **B** and **C**). **D**–**F**, Similar to **A**–**C**, but for those observed in the parvocellular MVN/PrH, in which axonal boutons showing *en passant* structures were dominantly observed (arrows in **E** and **F**). MVMC, mgnocellular NVN; MVPC, parvocellular MVN; PrH, prepositus hypoglossi nucleus; 4V, fourth ventricle. Scale bars, 200 μm (**A** and **D**) and 20 μm (**B**, **C**, **E** and **F**).

Then, we compared the structure of FL P-cell axonal boutons between the magnocellular MVN region and the parvocellular MVN/PrH region by higher-magnification LM. In the magnocellular MVN (dashed square in Fig 3A), the FL P-cell axonal boutons were relatively large and came in contact with somata or proximal dendrites, showing a “basket like structure” (arrowheads in Fig 3B and 3C), as observed in the previous studies [23,34]. In contrast, in the parvocellular MVN/PrH (dashed square in Fig 3D), the FL P-cell axons tended to have smaller varicosities, showing an “*en passant* structure” (arrows in Fig 3E and 3F). Such axonal boutons with *en passant* structures were rarely observed on the NeuN-positive somata of MVN neurons, suggesting the presence of axodendritic synaptic contacts in the parvocellular MVN/PrH.

**Fig 4:**
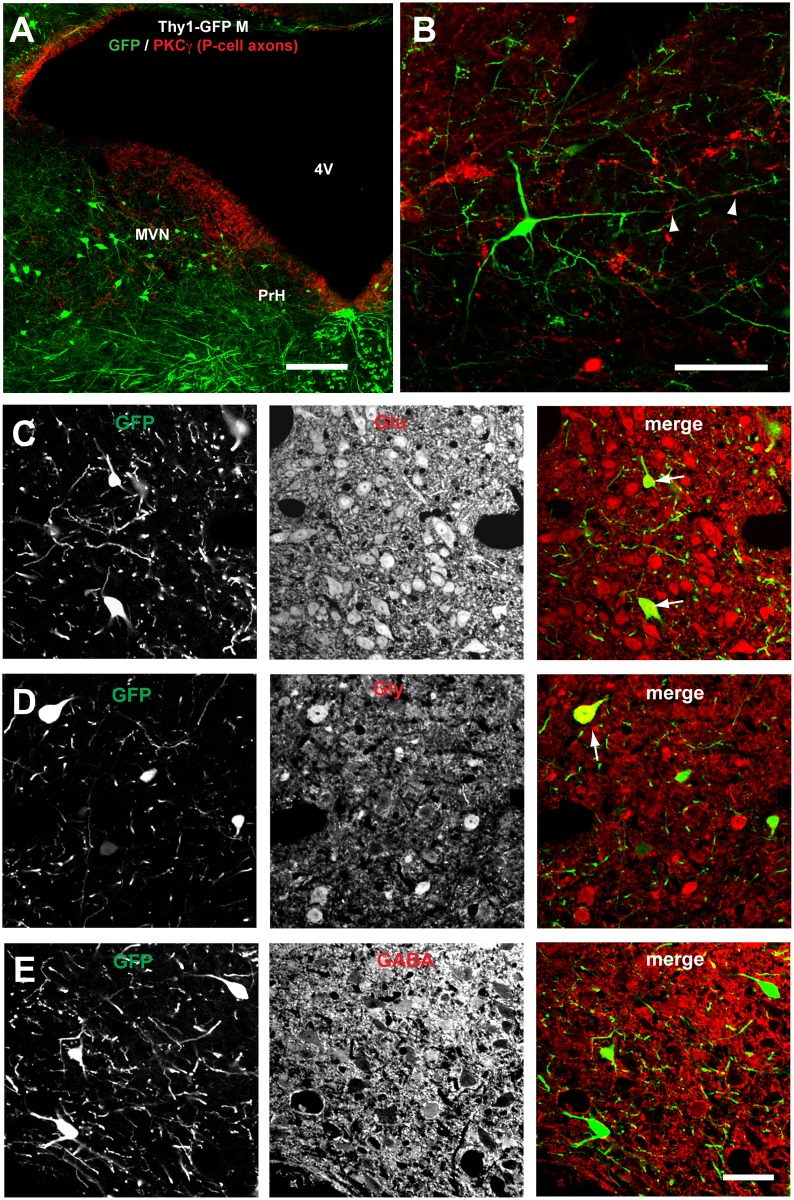
Neurons that receive FL P-cell projections on their distal dendrites in the parvocellular MVN/PrH are predominantly glutamatergic. **A** and **B**, Representative images of MVN/PrH neurons of Thy1-GFP M-line transgenic mouse that were stained with anti-GFP (green) and anti-PKCγ (red) antibodies. **C**, **D**, and **E**, Images of coronal sections of MVN/PrH stained with either one of three antibodies against neurotransmitters (anti-Glu, anti-Gly, and anti-GABA). The majority of GFP-labeled neurons were stained positive for anti-Glu antibody (**C**), and some of the remaining neurons were positive for anti-Gly antibody (**D**), but not positive for anti-GABA antibody (**E**). Scale bars, 200 μm (**A**), 100 μm (**B**), and 50 μm (**C**–**E**). GABA, γ-aminobutyric acid; Glu, glutamate; Gly, glycine.

To further analyze the features of synaptic contacts of P-cell axons on MVN dendrites, we used Thy1-GFP M-line transgenic mice that express GFP in only a small population of MVN neurons. The GFP-labeled neurons were evenly distributed throughout MVN in the sections obtained from Thy1-GFP M-line transgenic mice (Fig 4A and 4B). We traced the dendrites and somata of such GFP-labeled MVN neurons, and labeled the P-cell axons and their terminal boutons in eight mice using the antibody against the protein kinase Cγ-subunit (PKCγ), which is a marker for P-cells. The PKCγ immunoreactivity is known to be present only in P-cell axons, but not in other structures in MVN [35]. In the parvocellular MVN and PrH, anti-PKCγ antibody-labeled P-cell axon terminals were located on the thin distal dendrites, but rarely on the somata or proximal dendrites (Fig 4B, also see S1 Fig).

Because MVN is considered to be composed of neurons expressing several different neurotransmitters, we examined types of the neurotransmitters expressed in FL-targeted parvocellular MVN/PrH neurons by the double immunostaining using the anti-GFP antibody and either one of the three antibodies against neurotransmitters (anti-Glu, anti-Gly, and anti-GABA) in one Thy1-GFP M-line transgenic mouse by LM. Since neurotransmitters used in the FL-targeted manocelullar MVN neurons were already analyzed in a previous study [34], we focused on the parvocellular MVN/PrH. Thirty-three of 35 (94%) neurons examined were determined to be glutamatergic, whereas 6 of 24 (25%) and 0 of 22 (0%) neurons examined were determined to be glycinergic and GABAergic, respectively (Fig 4).

Then, we confirmed these observations by in-situ hybridization for vesicular glutamate transporter 2 (VGluT2) in the brain sections obtained from two Thy1-GFP M-line transgenic mice (S2 Fig), because it is known that the immunostaing using anti-Glu does not specifically label the somata of glutamatergic neurons. As previous two studies [36,37] consistently suggest that VGluT2 is expressed abundantly throughout glutamatergic MVN neurons, whereas vesicular transporter 1 (VGluT1) is expressed in a small portion of magnocellular MVN neurons, we examined the colocalization of GFP and VGluT2 in the parvocellular MVN/PrH. VGluT2 was expressed abundantly in both the parvocellular MVN and PrH, and 37 of 41 (90%) GFP-labeled neurons were positive for VGluT2 (S2 Fig). Thus, most of the parvocellular MVN/PrH neurons that receive direct FL P-cell projections on their distal dendrites seem to be glutamatergic.

### Ultrastructure of synapses between FL P-cells and parvocellular MVN/PrH neurons

**Fig 5:**
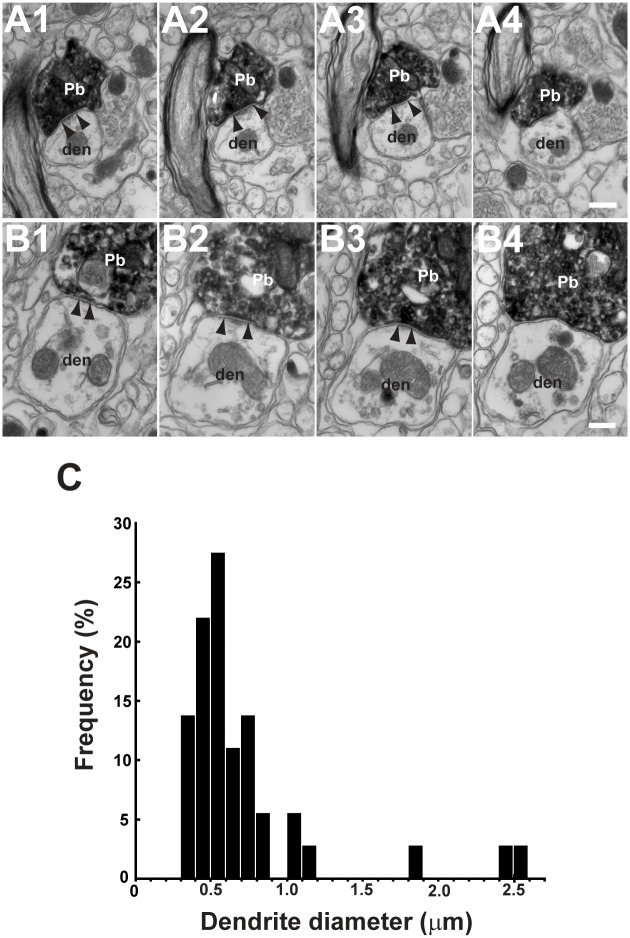
Axodendritic synapses between FL P-cells and parvocellular MVN/PrH neurons. **A1**–**A4**, **B1**–**B4**, Photographs of two examples of EM serial sections (interval, 0.14 μm) for FL P-cell axonal boutons forming symmetrical synapses on parvocellular MVN/PrH neurons. Edges of synapses were shown by arrowheads. **C**, Distribution of the mean diameters for post-synaptic dendrites. Note that FL P-cell axonal boutons formed synapses on relatively small diameter dendrites (mean, 0.73 μm; n = 40 dendrites from three mice). den, dendrite of MVN/PrH neuron; Pb, P-cell axonal bouton. Scale bars, 0.2 μm.

We analyzed by EM the synaptic structure of neurons in the border region between the parvocellular MVN and PrH, where P-cell axonal boutons were densely present. Consistent with the results of our LM study, all of the 49 GFP-labeled FL P-cell axonal boutons examined were in contact with thin dendrites, but not with somata, as reported in a previous EM study of deep cerebellar nuclei (DCN) of rats [38]. The GFP-labeled P-cell axonal boutons contained many flattened vesicles, showing the typical GABAergic symmetrical synaptic structure in which axonal boutons are in contact with dendrites or dendritic spines (arrowheads in Fig 5A1–5A3 and 5B1–5B3, also see S3 Fig). Most of such boutons contained a large number of synaptic vesicles and small mitochondria and were often surrounded or ensheathed by glial processes. As shown by previous EM studies of DCN [39,40], more than one synaptic contact was observed per bouton (data not shown in figures).

Next, to further determine whether the FL P-cell axonal boutons form synapses on distal dendrites or proximal dendrites in the parvocellular MVN/PrH, we measured the diameters of the target dendrites at each synapse (Fig 5C). The mean dendritic diameter at FL P-cell synaptic regions was 0.730 ± 0.077 μm (mean ± SEM; n = 40). Because previous studies of rats [38] and rabbits [41] consistently demonstrated that the diameter of the distal dendrites of neurons of lateral vestibular nucleus and PrH is less than 1.5–2 μm, we suggest that the FL P-cell axons may make synapses mainly on the distal dendrites of parvocellular MVN/PrH neurons.

**Fig 6:**
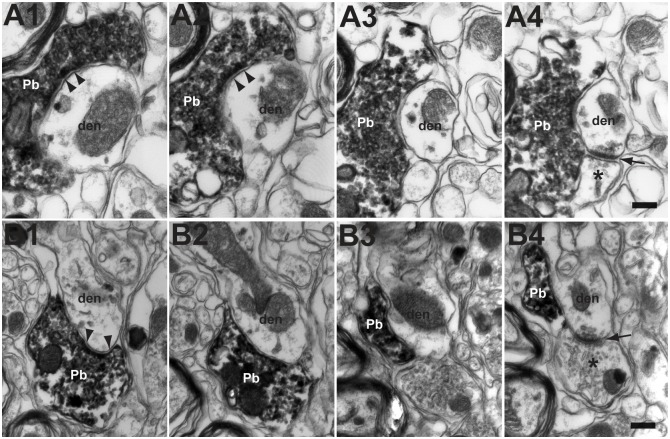
The location of FL P-cell axonal boutons and excitatory synapses on the dendrites of parvocellular MVN/PrH neurons. **A1**–**A4**, **B1**–**B4**, EM photographs of two examples of serial sections (interval, 0.21 μm) for the FL P-cell axonal boutons forming symmetrical synapses on MVN/PrH neurons. Note that the FL axonal boutons (arrow heads) were located close to the excitatory synapses on the same dendrites (arrows). * indicates excitatory synapses. den, dendrite of MVN/PrH neuron; Pb, P-cell axonal bouton. Scale bars, 0.2 μm.

Interestingly, FL P-cell axonal boutons were often located close to excitatory synapses as shown by EM [27%, 13 in 49 boutons; see the FL P-cell axonal inhibitory (symmetric) synapses indicated by arrowheads and the excitatory (asymmetric) synapses indicated by arrows in Fig 6]. The mean distance between a P-cell inhibitory synapse and a neighboring excitatory synapse on the parvocellular MVN/PrH dendrite was 0.136 ± 0.036 μm (n = 16). Moreover, the FL P-cell axons sometimes formed synapses directly on the spine-like dendritic protrusions that did not contain mitochondria (18%, 9 in 49 boutons examined; arrowheads in S3B Fig, also see S1 Fig). Such apposition of P-cell axonal boutons to dendritic spines was found in previous anatomical studies of cerebellar nuclei, lateral vestibular nucleus, and PrH of rats and cats [39, 42–44].

### Spatial relationship between FL P-cell axonal boutons and vestibular nerve axonal boutons in parvocellular MVN/PrH

The main excitatory inputs to MVN neurons are the primary vestibular nerve afferents. To determine the relationship between the vestibular nerve axonal boutons and the FL P-cell axonal boutons in the parvocellular MVN/PrH, we first injected GFP-expressing lentivirus into FL, and later injected BDA into the horizontal semicircular canal in three C57/BL6J mice.

**Fig 7:**
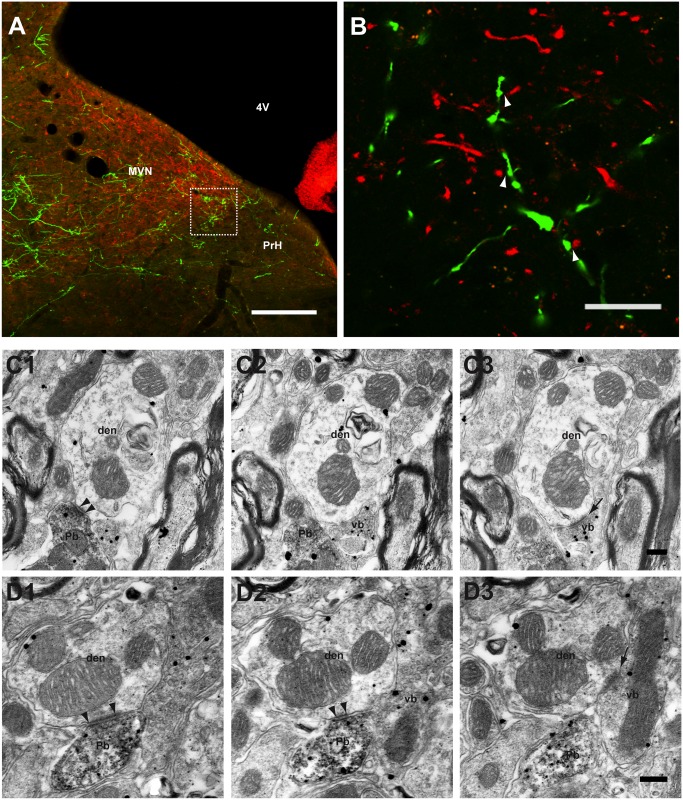
Distribution of vestibular nerve axonal boutons and FL P-cell axonal boutons in the parvocellular MVN/PrH. **A**, Low magnification image of MVN/PrH showing two distinct types of synaptic inputs. BDA was injected into the horizontal semicircular canal after the lentivirus injection into FL. FL P-cell axons and vestibular nerve axons were visualized by anti-GFP antibody (green) and Alexa 594 streptavidine (red), respectively. Note that the distribution of FL P-cell axons overlapped partially with that of the vestibular nerve axons in MVN/PrH. **B**, High magnification confocal image of the region marked by square in A. Some of the FL P-cell axonal boutons were closely located to those of the vestibular nerve axonal boutons (arrowheads). **C1**–**C3**, **D1**–**D3**, Two examples of serial EM photographs (interval, 0.14 μm in **C1**–**C3**, 0.21 μm in **D1**–**D3**) of MVN/PrH neuronal dendrites. GFP (FL P-cell axons) was detected by DAB reaction products, and BDA (vestibular nerve axons) was detected by silver-enhanced gold particles. Note that the FL P-cell axons formed symmetrical synapses (arrowheads), whereas the vestibular nerve axons formed asymmetrical synapses (arrows) on the same MVN neuronal dendrites. den, dendrite of MVN/PrH neuron; MVN, medial vestibular nucleus; Pb, P-cell axonal bouton; PrH, prepositus hypoglossi nucleus; Vb, vestibular nerve axonal bouton; 4V, fourth ventricle. Scale bars, 200 μm (**A**), 20 μm (**B**), and 0.2 μm (**C1**–**C3**, **D1**–**D3**).

In these mice, the BDA-labeled vestibular nerve axonal boutons were distributed throughout the ipsilateral MVN as shown by previous studies of cats [45,46]. They partially overlapped with the GFP-labeled FL axonal boutons (Fig 7A and 7B). Then, to examine the spatial relationship between these two types of boutons on the parvocellular MVN/PrH neurons, we carried out double-labeling immuno-EM study for GFP and BDA, which respectively produced DAB precipitates and silver-enhanced gold particles. The BDA-labeled vestibular nerve axonal boutons were located mainly on distal dendrites, but rarely on somata or proximal dendrites, showing excitatory synaptic profiles characterized by the presence of asymmetrical densities and round vesicles (Fig 7C3 and 7D3). In some MVN/PrH dendrites examined by EM, both the FL P-cell axonal boutons and the vestibular nerve axonal boutons were observed in the same sections. In such dendrites, the FL P-cell axonal inhibitory synapses were located close to the BDA-labeled vestibular nerve axonal excitatory synapses (Fig 7C1–7C3 and 7D1–7D3).

These EM observations were also supported by the LM study of the brain sections obtained from four Thy1-GFP M-line transgenic mice that received BDA injections in the horizontal semicircular canal. In these sections, the PKCγ-positive FL P-cell axonal boutons were in contact with the GFP-labled dendrites or spines, closely adjacent to the BDA-labeled vestibular nerve axonal boutons in the parvocellular MVN/PrH (S1A and S1B Fig). In summary, the FL P-cell axons mainly formed synapses on distal dendrites, often located close to the excitatory synapses of vestibular nerve axons in the parvocellular MVN/PrH.

## Discussion

In the present study, the distribution and structure of synapses between FL P-cells and MVN neurons were analyzed by lentivirus-mediated anterograde tracing of FL P-cells and BDA anterograde tracing of vestibular nerve axons in mice. The FL P-cell axons terminated in both the magnocellular MVN and parvocellular MVN/PrH regions, with a slight predominance in the parvocellular MVN/PrH. Moreover, the pattern of termination differed between the magnocellular MVN and parvocellular MVN/PrH. LM study demonstrated that FL P-cells mainly terminated on the somata and proximal dendrites of magnocellular MVN neurons, whereas both LM and EM studies demonstrated that the inhibitory synapses of FL P-cells and the excitatory synapses of vestibular nerve afferents were often located close to each other on the distal dendrites of parvocellular MVN/PrH neurons.

### Distribution of FL inhibitory inputs in MVN

Previous studies consistently demonstrated that a small number of MVN neurons receive direct P-cell projections from the middle zone of FL in mice [12,23], rabbits [13,21,47], cats [22], and monkeys [31,48]. These neurons are the magnocellular MVN and the parvocellular MVN, which respectively contain neurons of relatively large and small somata. The magnocellular MVN corresponds to the nucleus of ascending tract of Deiters in rabbits and cats [49,50] and the ventrolateral vestibular nucleus in monkeys [51].

Using the GFP-expressing VSVG pseudotyped lentiviral vector as an anterograde tracer, we showed that FL P-cell axonal boutons were distributed in both the magnocellular MVN and parvocellular MVN/PrH border regions in mice (Fig 2E–2G). However, previous two studies of mice [23,34] suggested that the FL P-cells project mainly to the magnocellular MVN and terminate on their somata and proximal dendrites. Because the authors of these previous studies used both electrophysiological recording and anatomical analysis of transgenic mice expressing tau-GFP fusion protein for studying the FL-targeted MVN neurons, it is conjectured that they mainly focused on the magnocellular MVN neurons and did not closely examine the parvocellular MVN/PrH neurons. Moreover, it was reported [52] that the cytosolic GFP signals at thin neurites are generally brighter than those in neurons expressing tau-GFP that was used in the study mentioned above.

### Two different types of FL-MVN synaptic structures

**Fig 8:**
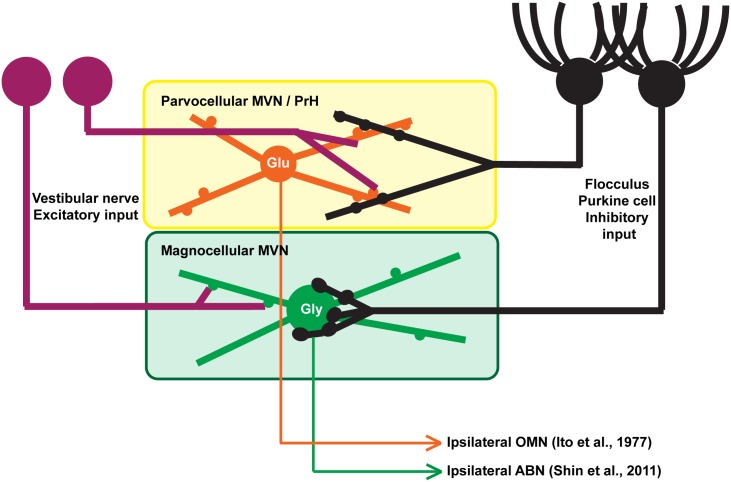
A putative scheme for the two different types of FL-targeted HVOR/HOKR-relaying neurons in mouse MVN. In magnocellular MVN, FL P-cell axons make synaptic contacts on the proximal dendrites and somata of neurons [23,34], Figs 2 and 3 in the present study), which are presumably inhibitory glycinergic neurons projecting to the ipsilateral abducens nucleus [34]. By contrast, in parvocelliar MVN/PrH, FL P-cells make synaptic contacts on the distal dendrites of neurons (Figs 2, 3 and 5), which are presumably excitatory glutamatergic (Fig 4 and S2 Fig) neurons projecting to the ipsilateral oculmotor nucleus [53]. Also see S1 Table. ABN, abducens nucleus; Glu, glutamatergic (excitatory) neuron; Gly, glycinergic (inhibitory) neuron; OMN, oculomotor nucleus.

The structures of FL P-cell axonal boutons were clearly different between the magnocellular MVN and parvocellular MVN/PrH (Fig 3). In the magnocellular MVN, FL P-cell axonal boutons were relatively large and predominantly in contact with the somata or proximal dendrites of MVN neurons, forming basket like structures. Such basket like structures were already reported by Sekirnjak et al. [23]. Moreover, Shin et al. [34] reported that FL P-cells project predominantly to the somata and proximal dendrites, and rarely (3%) to the proximal dendrites of the magnocellular MVN neurons, which was confirmed in the present study. They also reported that most (98%) of the FL-targeted magnocellular MVN neurons were inhibitory glycinergic and projected to the ipsilateral brainstem (Fig 8 and S1 Table).

On the other hand, the FL P-cell axonal boutons were shown to have varicosity-like structures in the parvocellular MVN/PrH by LM (Fig 3E and 3F), and axodendritic synaptic contacts were observed in distal dendrites by EM (Fig 5) in the present study. Because the FL-targeted parvocellular MVN/PrH neurons were determined to be predominantly glutamatergic (Fig 4 and S2 Fig), they are most likely the excitatory neurons projecting to the ipsilateral oculomotor nucleus [53] (Fig 8).

It is not known whether a single FL P-cell extends its axon collaterals to multiple MVN neurons, because our tracing method labeled a large number of P-cells in the medioventral FL (Figs 1 and 2). Single-cell tracing studies (e.g., [54]) are required to answer this question.

### FL projection to PrH

In previous studies, FL-PrH projections have been demonstrated in rats [55], rabbits [56], and cats [30,44], but not in monkeys [31,48]. In a previous study of mice [12], FL-PrH projections were not confirmed, because it was observed after only one FL BDA injection with labeling of ventral PFL. However, in the present study, FL-PrH projections were observed in 5 of 13 FL lentivirus injections, in which the ventral PFL was not labeled with GFP (Table 1). Thus, FL-PrH projections are conserved among mice, rats, rabbits, and cats.

In PrH, FL P-cell axonal boutons were observed in the border between the parvocellular MVN and PrH at their rostral poles, which was referred to as the marginal zone (MZ) in a study of monkeys and cats by Langer et al. (1986) [57]. A study of cats by Yingcharoen et al. (1983) demonstrated that MZ abundantly contains glutamate decarboxylase (GAD)-positive fibers [44]. Because GAD is a marker of GABAergic transmission, this study was consistent with the present study showing that a large number of FL P-cell axons projected to MZ. Since an HRP injection study demonstrated that MZ contains neurons projecting to contralateral abducens nucleus [57], some of the parvocellular MVN/PrH, which are not targeted by FL P-cells may project to the contralateral abducens nucleus.

### Comparison of FL-targeted MVN neurons with DCN neurons

As shown in Fig 8, the FL-targeted MVN neurons have different sizes of their somata with different types of neurotransmitters, i.e., magnocellular glycinergic neurons and parvocellular/PrH glutamatergic neurons (also see [58,59]). DCN are also composed of neurons with different sizes of their somata and different types of neurotransmitters. Glycinergic neurons with small somata are considered to be inhibitory interneurons, whereas those with large somata project to the brainstem [60]. GABAergic neurons with small somata project to the inferior olive, and glutamatergic neurons with large somata project to the red nucleus, thalamus, or brainstem. Previous LM studies of rats suggest that glutamatergic DCN neurons receive P-cell axons mainly on their somata [61–63]. However, an EM study of rats by De Zeeuw and Berrebi [38] demonstrated that P-cells predominantly project to non-GABAergic DCN neurons: 8% of P-cell axons terminate on their somata, 29% on their proximal dendrites, and 42% on their distal dendrites, which is analogous to the results of the present study. Thus, P-cells form both axosomatic and axodendritic synapses in both DCN and FL-targeted MVN.

### Functional implication of present findings

The unique finding of the present study is that a large number of FL P-cells form their inhibitory synapses on the distal dendrites of parvocellular MVN/PrH neurons, frequently located close to the excitatory synapses of vestibular nerve axons. Such a close spatial relationship between the inhibitory P-cell synapses and the excitatory synapses on MVN neuronal dendrites were not reported previously. Below, we discuss its possible functional significance.

It has been considered for long that inhibitory neurons mainly form synapses near somata to directly shunt the action potentials generated at the somata or initial axonal segments (e.g., [64,65]). The pattern of FL P-cell innervation on magnocellular MVN neurons is consistent with this traditional view of inhibitory synapses. Long-term depression of parallel fiber-Purkinje cell synapses, which is suggested to underlie HVOR or HOKR adaptation [6,7,19,20], should weaken the shunting action of FL P-cells on magnocellular MVN neurons. Because the FL-targeted MVN neurons relay both HVOR and HOKR signals (e.g., [2]), the weakened shunting action would affect both HVOR and HOKR. The enhanced HVOR gain after HOKR training observed in previous studies of mice [8] and rabbits [66] may be induced by such a mechanism.

It has been demonstrated that inhibitory neurons of the hippocampus or cerebral cortex sometimes form synapses on distal dendrites or even on spines. For example, a group of cerebral cortical GABAergic interneurons terminate on the dendritic spines of pyramidal neurons that mainly receive thalamocortical excitatory inputs in rats or mice [67,68]. In such pyramidal neurons, the inhibitory synaptic inputs are considered to shunt the thalamocortical excitatory synaptic inputs on the same dendrites (also see [65]). Thus, we suggest that the FL P-cell axonal synapses on distal dendrites or spines may directly influence the signal transmission of excitatory synapses nearby in parvocellular MVN/PrH neurons. Interestingly, a model study [69] suggests that the input- spike-timing-dependent plasticity [70–72] occurs more efficiently in MVN neurons, when FL P-cells and vestibular nerve axons innervate the same distal dendrites of MVN neurons.

Our previous studies of mouse HOKR demonstrated that the blockade of protein synthesis during training by bilateral floccular applications of anisomycin or actinomycin D [9], or the shutdown of spontaneous FL P-cell activity during post-training periods by bilateral floccular applications of muscimol [73] impaired the formation of long-term memory of adaptation in MVN. Moreover, two electrophysiological experiments using rabbit in-vivo [74] and mouse brainstem slice [75] preparations consistently suggested that the peptide hormone, motilin, may be coreleased with GABA from P-cell axon terminals in vestibular nuclei to modulate the excitability of postsynaptic nuclear neurons. On the basis of these observations, we suggest that some proteins or peptides, which are synthesized in FL P-cells during training, may be transferred to MVN through the axonal transport, and released to induce the plasticity in excitatory synapses on MVN neurons [70–72,75], which underlies the long-term memory of adaptation [8]. Although further studies are necessary to examine whether such a scenario is possible or not, the very short distance between the inhibitory synapses and the excitatory synapses (mean, 0.136 μm; Fig 6) on the distal dendrites of parvocellular MVN/PrH neurons may be favorable for the diffusion of such proteins from P-cell inhibitory synapses to excitatory synapses.

Finally, a recent model study [76] has suggested that FL P-cells have an important role in plasticity induction at the synapses between mossy fibers and MVN/DCN neurons [70–72] during post-training periods. Thus, the apposition of the FL P-cell axonal synapses and vestibular nerve axonal synapses on parvocellular MVN/PrH neuronal dendrites may provide a morphological basis for the consolidation of memory of cerebellovestibular motor learning.

## Supporting Information

S1 Fig Distribution of P-cell axonal boutons on the parvocellular MVN/PrH neuronal dendrites examined by LM. **A** and **B**, Fluorescent images of MVN neuronal dendrites in Thy1-GFP M-line transgenic mice. Vestibular nerve axons were labeled by BDA, which were visualized using Alexa 647-streptavidine (blue). MVN neurons were stained with anti-GFP antibody (green). P-cells axons were labeled by anti-PKCγ antibody (red). Note that the inhibitory (arrowheads) and excitatory (arrows) axonal boutons apposed on the same dendrites. **C**, **D**, and **E**, Parvocellular MVN/PrH neurons of Thy1-GFP M-line transgenic mice stained by anti-GFP (green), anti-PKCγ (red), and anti-synaptophysin (blue) antibodies. P-cell axonal boutons often targeted dendritic spines of Parvocellular MVN/PrH neurons, partially overlapping with synaptophysin clusters (arrowheads). Scale bars, 2 μm (**A** and **B**) and 1 μm (**C**–**E**).
(TIF)

S2 Fig Double in-situ hybridization of the parvocellular MVN/PrH neurons for GFP and VGluT2 in Thy1-GFP M-line mice. **A–C** and **D–F**, Expression of GFP and VGluT2 in the parvocellular MVN/PrH revealed by double in-situ hybridization experiments. Note that the majority of GFP-labeled neurons (green) with DAPI (blue) signals in the parvocellular MVN/PrH were overlapped with VGluT2 (red) signals (arrowheads), suggesting that the GFP-labeled parvocellular MVN/PrH neurons were predominantly glutamatergic. Scale bar, 100 μm.
(TIF)

S3 Fig Dendritic spines in Golgi-stained preparation and immuno-EM section. **A**, Camera lucida drawing of two representative Golgi-stained parvocellular MVN/PrH neurons. Spines were distributed throughout the dendritic branches of neurons. **B**, Examples of the dendritic spines innervated by P-cell axonal boutons, showing symmetrical synapses (arrowheads). Pb, P-cell axonal bouton; sp, spine. Scale bars, 100 μm (**A**) and 0.1 μm(**B**).
(TIF)

S1 Table Properties of the magnocellular MVN (Sekirnjak et al., 2003; Shin et al., 2011) and parvocellular MVN/PrH (present study) neurons innervated by FL P-cells. (DOC)
