# Supplementary figures and images for: Distribution and Structure of Synapses on Medial Vestibular Nuclear Neurons Targeted by Cerebellar Flocculus Purkinje Cells and Vestibular Nerve in Mice: Light and Electron Microscopy Studies
Source: PLoS One. 2016 Oct 6;11(10):e0164037. doi: 10.1371/journal.pone.0164037 (PMC5053601; doi:10.1371/journal.pone.0164037)

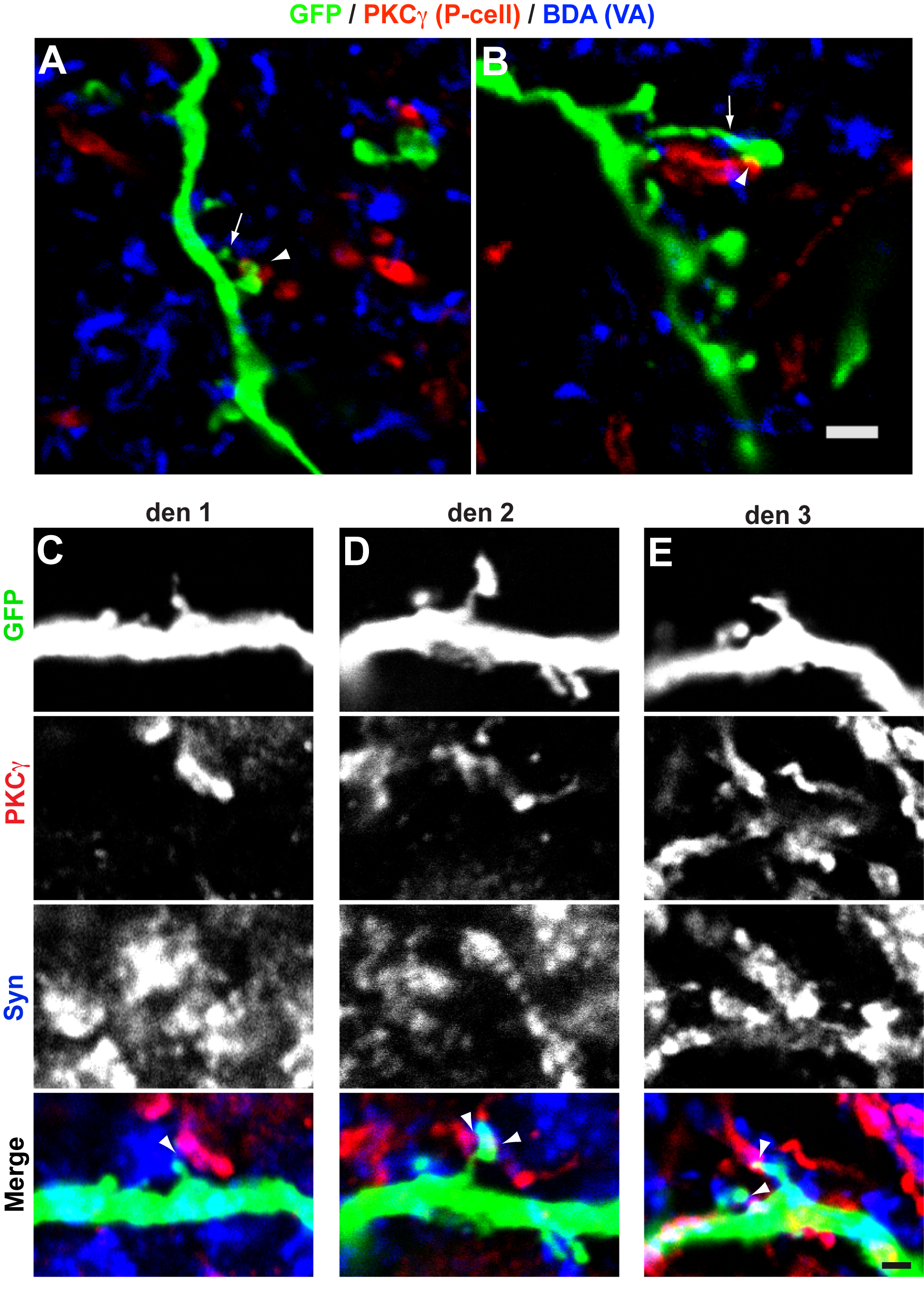

Supplement: S1 Fig — A and B, Fluorescent images of MVN neuronal dendrites in Thy1-GFP M-line transgenic mice. Vestibular nerve axons were labeled by BDA, which were visualized using Alexa 647-streptavidine (blue). MVN neurons were stained with anti-GFP antibody (green). P-cells axons were labeled by anti-PKCγ antibody (red). Note that the inhibitory (arrowheads) and excitatory (arrows) axonal boutons apposed on the same dendrites. C, D, and E, Parvocellular MVN/PrH neurons of Thy1-GFP M-line transgenic mice stained by anti-GFP (green), anti-PKCγ (red), and anti-synaptophysin (blue) antibodies. P-cell axonal boutons often targeted dendritic spines of Parvocellular MVN/PrH neurons, partially overlapping with synaptophysin clusters (arrowheads). Scale bars, 2 μm (A and B) and 1 μm (C–E). (TIF) [file pone.0164037.s001.tif]

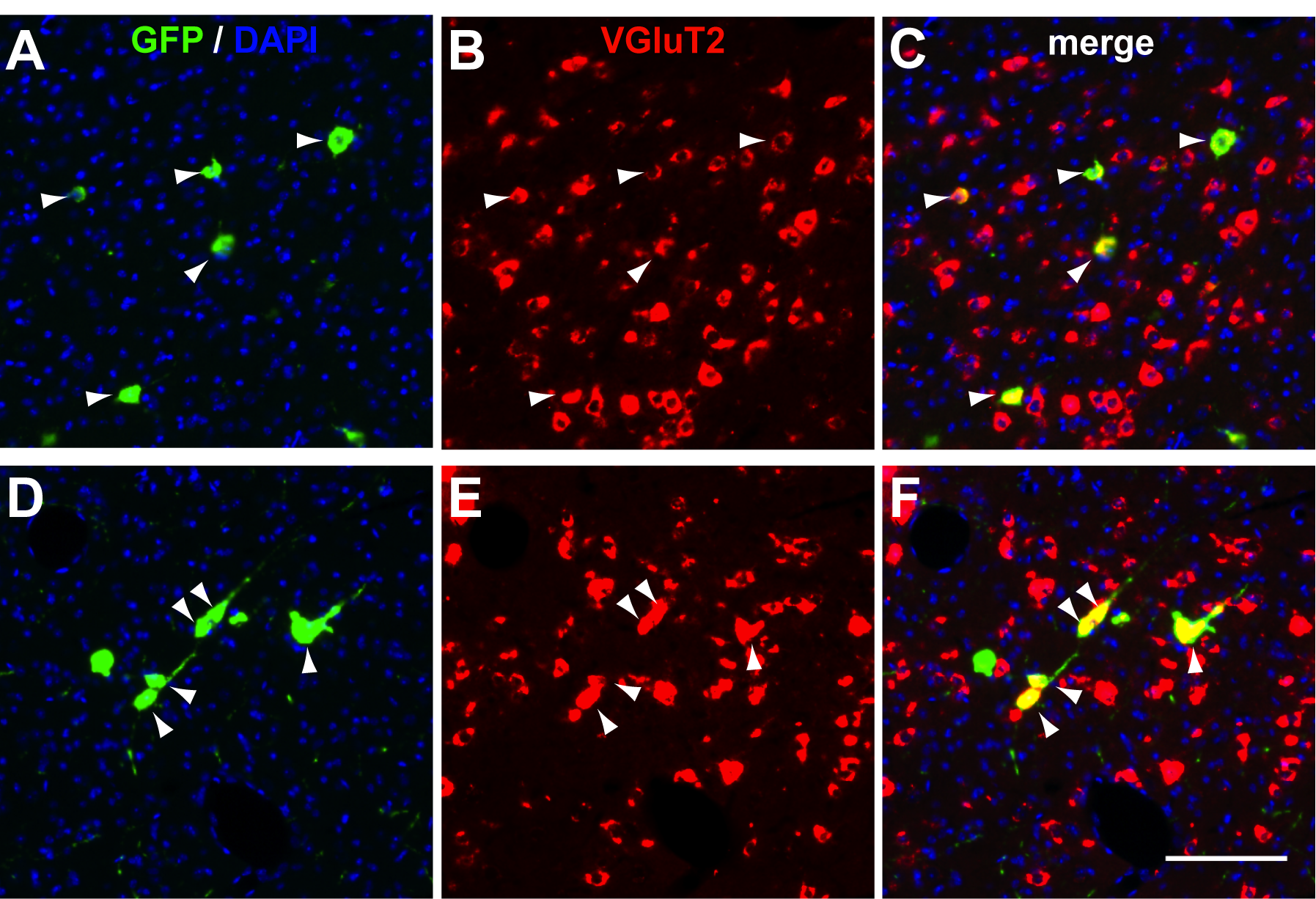

Supplement: S2 Fig — A–C and D–F, Expression of GFP and VGluT2 in the parvocellular MVN/PrH revealed by double in-situ hybridization experiments. Note that the majority of GFP-labeled neurons (green) with DAPI (blue) signals in the parvocellular MVN/PrH were overlapped with VGluT2 (red) signals (arrowheads), suggesting that the GFP-labeled parvocellular MVN/PrH neurons were predominantly glutamatergic. Scale bar, 100 μm. (TIF) [file pone.0164037.s002.tif]

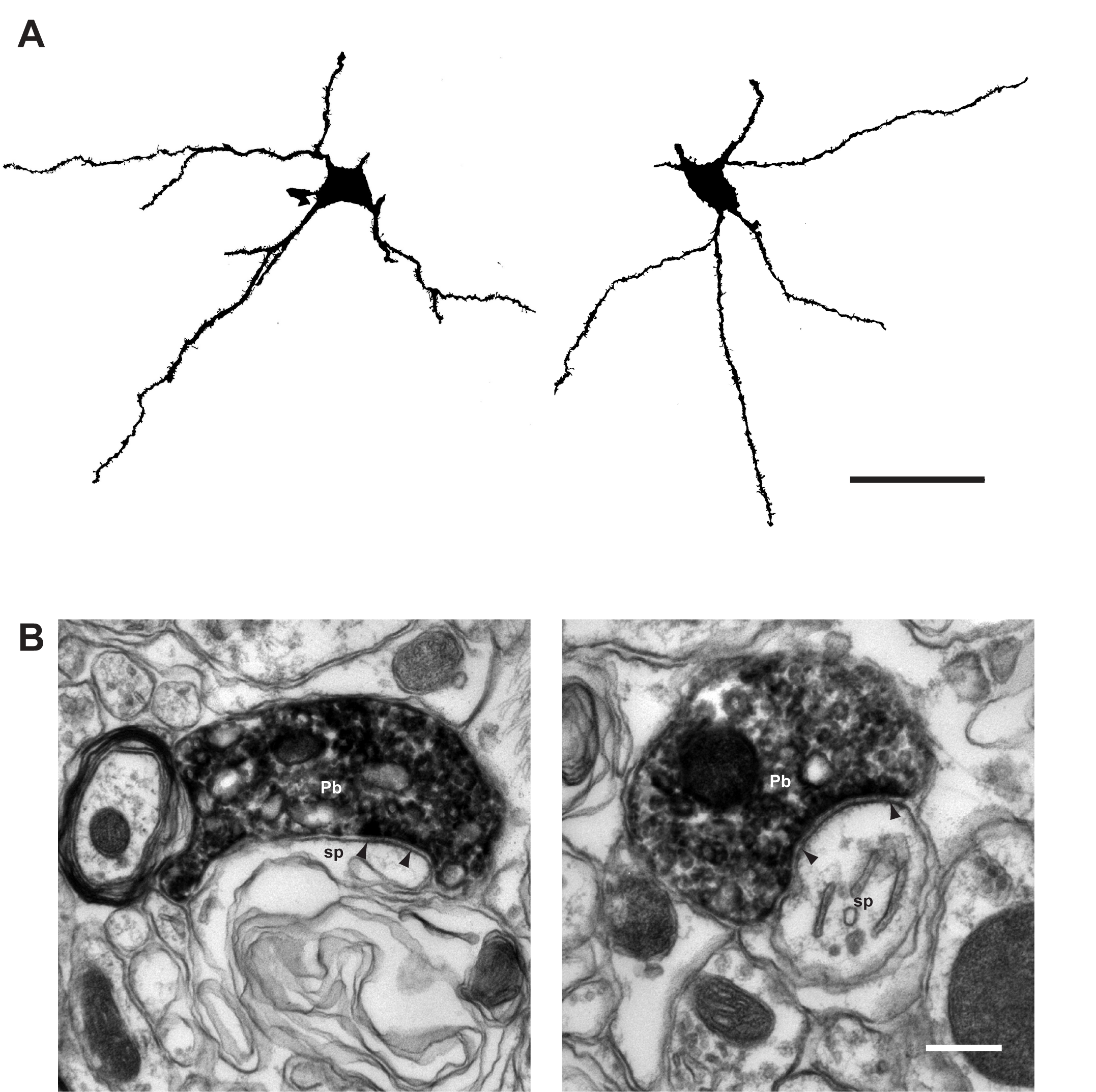

Supplement: S3 Fig — A, Camera lucida drawing of two representative Golgi-stained parvocellular MVN/PrH neurons. Spines were distributed throughout the dendritic branches of neurons. B, Examples of the dendritic spines innervated by P-cell axonal boutons, showing symmetrical synapses (arrowheads). Pb, P-cell axonal bouton; sp, spine. Scale bars, 100 μm (A) and 0.1 μm(B). (TIF) [file pone.0164037.s003.tif]
