# Supplementary material for: Distribution and Structure of Synapses on Medial Vestibular Nuclear Neurons Targeted by Cerebellar Flocculus Purkinje Cells and Vestibular Nerve in Mice: Light and Electron Microscopy Studies
Source: PLoS One. 2016 Oct 6;11(10):e0164037. doi: 10.1371/journal.pone.0164037 (PMC5053601; doi:10.1371/journal.pone.0164037)
Supplement: S1 Table — (DOC) [file pone.0164037.s004.doc]

**S1 Table. Properties of the magnocellular (Sekirnjak et al., 2003; Shin et al., 2011) and parvocellular /PrH (present study) MVN neurons innervated by FL P-cells**.

|  | Major location of FL P-cell synapses | Transmitter  used | Percentage of neurons examined  (Mouse strain and antibodies/in-situ probe used for histology) | Output |
| --- | --- | --- | --- | --- |
| Magnocellular  MVN | Somata /proximal  dendrites | Glu  Gly  GABA | Not known  98% (GlyT2-GFP, anti-calbindin)  95% (Thy1-YFP-16, anti-calbindin)  7% (GAD67-GFP, anti-calbindin)  0% (GAD65-GFP, anti-calbindin) | Not specified  Ipsi ABN  Not known |
| Parvocellular/PrH  MVN | Distal dendrites | Glu  Gly  GABA | 94% (Thy1-GFP M-line, anti-PKCγ, anti-Glu)  90% (Thy1-GFP M-line, anti-PKCγ, VGluT2 in-situ probe  25% (Thy1-GFP M-line, anti-PKCγ, anti-Gly)  0% (Thy1-GFP M-line, anti-PKCγ, anti-GABA) | Ipsil OMN [1]  Ipsi ABN [1]  Not known |

In the study of Shin et al. (2011), the neurotransmitters used in MVN neurons were determined by anti-calbindin immunostaining for P-cell axonal boutons in the transgenic mice expressing GFP in neurons containing a particular type of neurotransmitter. Meanwhile, in the present study, they were determined by anti-PKCγ, anti-Glu, anti-Gly, and anti-GABA immunostaining, and in-situ hybridization for VGluT2 in Thy1-GFP M-line mice which express GFP in some MVN neurons. See text for detail. GABA, γ-amino butyric acid; GAD67-GFP, GAD67-GFP knock-in mice expressing GFP in a subset of GABAergic neurons [2]; GAD65-GFP, GAD65-GFP mice expressing GFP under the GAD65 promoter in a subset of GABAergic neurons [3]. Glu, glutamate; Gly, glycine; GlyT2-GFP, mice expressing GFP under glycine transporter 2 promoter [4]; Ipsi, ipsilateral; Thy1-YFP-16, mice expressing YFP (yellow fluorescent protein) under thy1 promoter, which labels both glycinergic and glutamatergic neurons [5,6]; VGluT2, vesicular glutamate transporter 2.

1. Ito M, Nisimaru N, Yamamoto M. Specific patterns of neural connections involved in the control of rabbit’s vestibulo-ocular reflexes by the cerebellar flocculus. J Physiol (Lond) 1977: 265; 833-854. PMID: 300801.

2. Tamamaki N, Yanagawa Y, Tomioka R, Miyazaki J, Obata K, Kaneko T. Green fluorescent protein expression and colocalization with calretinin, parvalbumin, and somatostatin in the GAD67-GFP knock-in mouse. J Comp Neurol 2003: 467:60 –79. PMID: 14574680.

3. Lo´ pez-Bendito G, Sturgess K, Erde´lyi F, Szabo´ G, Molna´r Z, Paulsen O. Preferential origin and layer destination of GAD65-GFP cortical interneurons. Cereb Cortex 2004: 14; 1122–1133. PMID: 15115742.

4.. Zeilhofer HU, Studler B, Arabadzisz D, Schweizer C, Ahmadi S, Layh B, Bo¨sl MR, Fritschy JM. Glycinergic neurons expressing enhanced green fluorescent protein in bacterial artificial chromosome transgenic mice. J Comp Neurol 2005: 482; 123–141.

PMID: 15611994.

5. Feng G, Mellor RH, Bernstein M, Keller-Peck C, Nguyen QT, Wallace M, Nerbonne JM, Lichtman JW, Sanes JR. Imaging neuronal subsets in transgenic mice expressing multiple spectral variants of GFP. Neuron: 2000; 28:41–51. PMID: 11086982.

6. Bagnall MW, Zingg B, Sakatos A, Moghadam SH, Zeilhofer HU, du Lac S. Glycinergic projection neurons of the cerebellum. J Neurosci 2009: 29:10104 –10110. PMID: 19675244.
